# Five things the intensivist cannot forget in the management of invasive candidiasis

**DOI:** 10.62675/2965-2774.20260217

**Published:** 2026-01-28

**Authors:** José-Artur Paiva, Cidália Pina-Vaz, José Manuel Pereira

**Affiliations:** 1 Universidade do Porto Faculdade de Medicina Centro Hospitalar Universitário de São João Porto Portugal Intensive Care Medicine Service, Centro Hospitalar Universitário de São João, Faculdade de Medicina, Universidade do Porto - Porto, Portugal.; 2 Universidade do Porto Faculdade de Medicina Department of Medicine Porto Portugal Department of Medicine, Faculdade de Medicina, Universidade do Porto - Porto, Portugal.

## INTRODUCTION

Intensivists are increasingly faced with the suspicion and diagnosis of invasive candidiasis, as critically ill patients more often have risk factors for this disease. In this paper, we present five things that the intensivist must know to address this challenge. These items, which include tools available for diagnosis, main principles of therapy (drug and dose) and the importance of source control, were chosen because of their relevance for a pragmatic clinical approach to the patient.

## CHANCES OF ENCOUNTERING A RESISTANT *CANDIDA* SPECIES ARE HIGH

The incidence of invasive candidiasis is increasing because of the increase in the at-risk population.^(1)^ Although *C. albicans* remains the most common *Candida* species responsible for invasive infection, an increasing proportion of invasive candidiasis cases are caused by nonalbicans species, particularly *C. glabrata* and *C. parapsilosis*, as well as *C. tropicalis* and *C. krusei*. These five species account for more than 90% of all infections.^(1)^ Due to this shift to non-albicans species of *Candida* that more often present intrinsic resistance to antifungal drugs - such as C. *lusitaniae* to amphotericin B, *C. krusei* to fluconazole, *C. glabrata* dose-dependency to candins and *C. parapsilosis* reduced susceptibility to candins - and due to the emergence of acquired resistance, chances of encountering a resistant *Candida* are rising.^(2)^

Exposures to azoles and candins are the main drivers of acquired resistance to each of these drug families. The inhibition of glucan synthase by echinocandins weakens the fungal cell wall and causes significant cell wall stress that induces a variety of adaptive fungal protective mechanisms.^(3)^ These responses create a subpopulation of drug-tolerant persister cells with elevated minimum inhibitory concentrations (MICs) to echinocandins that do not induce therapeutic failure but can progress to higher-level resistance through the formation of stable FKS mutations.^(4)^

Multidrug-resistant (MDR) *Candida*, meaning an isolate non-susceptible to at least one agent in at least two antifungal classes, is a growing phenomenon, usually involving species such as *C. glabrata, C. parapsilosis or C. krusei* with previous intrinsic resistance to one drug.^(3)^

*C. auris* shows resistance to fluconazole in most cases, variable susceptibility to other azoles, and resistance to amphotericin B in approximately 35% of cases and to echinocandins in 5 - 10% of cases; moreover, half of *C. auris* isolates are MDR.^(5)^ Because breakthrough fungemia (defined as persistent fungemia after 3 days of antifungal treatment) and microbiological recurrence at 60 days are common, repeated blood cultures and MIC testing are paramount in *C. auris* infections.^(6)^

## ADEQUATE DIAGNOSIS IS A FUNDAMENTAL STEP FOR PROPER MANAGEMENT

Invasive candidiasis demands prompt and accurate diagnosis. Traditional culture-based methods are slow. Blood cultures—despite being the gold standard—can take several days to yield results and are negative in up to 50% of cases.^(7)^ Mass spectrometry allows for species identification directly from positive blood cultures within minutes using rapid extraction methods.^(8)^ Molecular identification panels applied directly to blood cultures offer fast results, but they are limited by incomplete pathogen databases and lower positive predictive values.^(7)^ Real-time polymerase chain reaction (PCR) assays directly from whole blood (without culture) are even more sensitive than blood cultures for difficult-growing microorganisms, use low volumes of blood and have a time-to-result of less than 3 hours. They serve as excellent "rule-out" tests for bloodstream infections with high negative predictive rates.^(9)^

Rapid susceptibility tests (ASTs), which are fundamental for bacteria,^(10)^ do not yet cover antifungal susceptibility. Molecular techniques aimed at detecting resistance mechanisms are hindered by an incomplete understanding of fungal resistance and inconsistencies between genotypic and phenotypic results. As such, rapid phenotypic antifungal susceptibility testing is urgently needed.

A promising approach, currently available only for bacteria, is FASTinov's flow cytometry-based AST, which is growth-independent and delivers results within two hours.^(11)^

The integration of rapid identification with fast AST has the potential to reduce empirical therapy and antifungal pressure.

Serum β-D-glucan (BDG) testing for the presumed diagnosis of IC is recommended, but the initiation of antifungal therapy should not be solely based on positive BDG, as the causes of false positivity are numerous.^(12)^ In conjunction with clinical parameters and in the context of antifungal stewardship (AFS), the use of BDG to decide early therapy is moderately supported by guidelines; however, some studies suggest a relevant proportion of false-negative results.^(12)^

## ANTIFUNGAL PHARMACOKINETICS/PHARMACODYNAMICS MUST BE CONSIDERED

A recent pharmacokinetics/pharmacodynamics (PK) study comprising critically ill patients prescribed antifungal agents^(13)^ revealed that there was considerable PK variability, that subtherapeutic exposures were present and that standard dosing was often insufficient for those pathogens with higher MICs. High doses may benefit patients at risk of underexposure but could also increase the risk of toxicity. This is salient for antifungals such as voriconazole that have a defined toxicity threshold. These findings underscore the importance of therapeutic drug monitoring for antifungal therapy in critically ill patients.^(3,14)^

**Figure 1:**
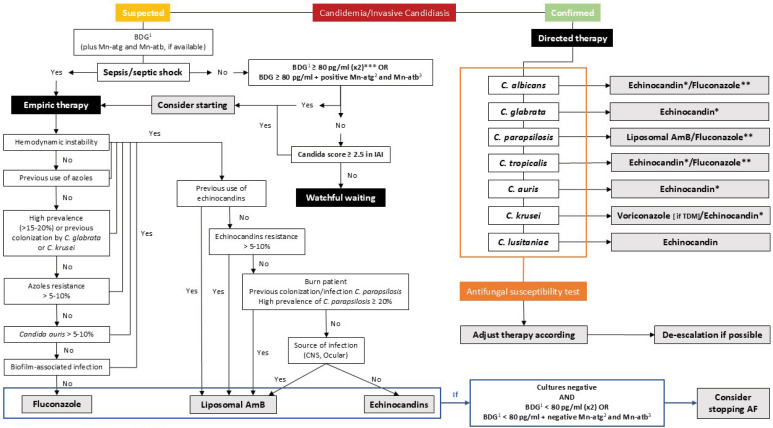
Treatment algorithm for suspected and confirmed invasive candidiasis. * Consider liposomal AmB as na alternative if echinocandin resistance > 5 - 10%; ** consider fluconazole as na alternative if hemodynamic stability and azoles resistance < 5%; *** to increase specificity consider ≥ 200pg/mL

BDG - B-D-glucan; Mn-atg - mannan antigen; Mn-atb - mannan antibody; AF - antifungal therapy. IAI - intrabdominal infection; TDM - therapeutic drug monitoring

Voriconazole is both a substrate and an inhibitor of CYP2C19 and CYP3A4. Allelic polymorphisms in CYP2C19 may result in rapid or slow metabolism of voriconazole, with significant variation in plasma levels.^(15)^ Factors affecting voriconazole PK include age, weight, dose and formulation, liver function, ethnic origin, systemic inflammation, the use of CYP2C19- and CYP3A-interacting medications, diet, antacids and proton pump inhibitors.^(15)^ Drug–drug interactions are numerous, and immunosuppressants and antiepileptics deserve special attention.^(3)^ Monitoring voriconazole through levels, between Days 2 and 5 of therapy should be performed in all patients taking the drug and is mandatory in children, obese patients, patients with interacting drugs, patients with altered organ function, patients on extracorporeal circuits or patients with suspected drug toxicity.^(3,14)^

Echinocandins share similar spectra of antifungal activity; however, each agent differs in its metabolic pathway, resulting in different half-lives, drug interactions and dosing strategies. They are distributed well into tissues, including the lungs, liver, and spleen. However, there is minimal penetration into the central nervous system and eye, due to their high protein binding and large molecular weight, and low penetration to heart and urinary tract. Candins, unlike azoles, are effective in treating biofilm-associated infections, although higher doses have been suggested.^(16)^ In critically ill patients, they are the first-line empirical therapy for candidemia, intra-abdominal candidiasis (other than peritoneal dialysis) and *Candida* endocarditis.^(12)^ Liposomal amphotericin B (L-AmB) is the first-line treatment for central nervous system candidiasis. L-AmB and azoles may be used for endophthalmitis.^(12)^

## ANTIFUNGAL STEWARDSHIP IS A NECESSARY TASK

Fungal resistance, driven by antifungal exposure, is becoming a serious threat, as antifungals increase the risk of drug toxicity and drug interactions. In fact, up to 50% of antifungal prescriptions are suboptimal or inadequate, and as the discontinuation of echinocandin and azole treatment leads to the disappearance of FKS alteration, the implementation of antifungal stewardship (AFS) is necessary and has the potential to be useful both on an individual basis and on an ecological basis.^(17)^ The AFS team should include fungal infection experts; promote the use of bundles of care; facilitate access to timely and adequate diagnosis; use education, auditing and "handshake" rounds at the bedside; be supported by local surveillance systems, including cumulative antifungal susceptibility reports; and define institutional and patient-level objectives with feedback to prescribers.^(17)^ Evidence suggests that AFS interventions can improve bundle compliance, decrease antifungal consumption, reduce adverse events and drug–drug interactions, ensure correct dosing and promote de-escalation or early cessation of empirical therapy.^(18)^

## EARLY TREATMENT, SOURCE CONTROL AND MANAGEMENT PERSONALIZATION IMPROVE OUTCOMES

A personalized treatment strategy improves efficacy, safety and outcome and limits antifungal overuse, slowing resistance development.

Early initiation of antifungal therapy is associated with improved survival.^(17)^ Therefore, empirical (started with only clinical suspicion of fungal infection) or preemptive (based on positive laboratory or radiological findings suggestive of fungal infection but without definitive proof) antifungal therapy is often used. Although echinocandins are generally considered first-line therapies because of their favorable safety profile, broad spectrum and limited drug interactions,^(12,16)^ host factors such as hemodynamics and immune status, organ dysfunction and drug interactions influence antifungal selection and dosing. The site of infection, previous use of antifungals, local susceptibility patterns, previous colonization and risk of biofilm formation should also be considered.

Personalized regimens based on therapeutic drug monitoring should be considered for triazoles and echinocandins in populations at risk of low or high drug exposure.^(12)^ De-escalation of antifungal therapy to a targeted regimen (an antifungal drug specifically chosen to treat a known fungal pathogen on the basis of laboratory identification) with an azole is appropriate when susceptibility is confirmed and when the patient is clinically stable.^(16)^

Prompt source control, including central venous catheter removal, is mandatory.^(12,16)^ Fourteen days of antifungal therapy from the first day of persistently negative blood cultures (three consecutive negative blood cultures) and resolution of symptoms are recommended.^(12,16)^ However, prolonged therapy may be necessary in patients with deep-seated candidiasis or inadequate source control, whereas shorter courses may be sufficient in immunocompetent patients with rapidly cleared candidemia and no metastatic complications. The integration of biomarkers and advanced imaging helps monitor response and guide treatment discontinuation. In patients with suspected (but not proven) invasive candidiasis, empirical antifungal therapy should be stopped if blood cultures and culture specimens from suspected infectious foci are negative, particularly if biomarkers are also negative^(16)^

## Data Availability

The contents will be made available at the time of publication of the article.
